# Multi-focused psychosocial residential rehabilitation interventions improve quality of life among cancer survivors: a community-based controlled trial

**DOI:** 10.1186/s12967-018-1618-0

**Published:** 2018-09-06

**Authors:** Xuefen Chen, Xiaohuan Gong, Changhong Shi, Li Sun, Zheng Tang, Zhengping Yuan, Jiwei Wang, Jinming Yu

**Affiliations:** 1 Key Lab of Public Health Safety of Ministry of Education and Key Lab of Health Technology Assessment of Ministry of Health, School of Public Health, Fudan University, No. 130 Dongan RD, Xuhui District, Shanghai, Zip code: 200032 China; 2 School of Public Health, Shanghai University of Traditional Chinese Medicine, No. 1200 Cailun Rd, Pudong New Area, Shanghai, Zip code: 201203 China; 3 Shanghai Cancer Rehabilitation Center, No. 164 Zhengning Rd 405 Nong, Shanghai, Zip code: 200050 China

**Keywords:** Cancer survivors, Psychosocial intervention, Rehabilitation, Quality of life

## Abstract

**Background:**

Even though multi-focused psychosocial residence rehabilitation intervention (MPRRI) programs are widely implemented by the Shanghai Cancer Rehabilitation Club, these programs have not been rigorously evaluated. In this study, we evaluated the effects of a 21-day MPRRI program, on the quality of life (QoL) among cancer survivors.

**Methods:**

A total of 388 cancer patients were enrolled to either receive the 21-day MPRRI (n = 129) intervention or a waiting-list comparison (WLC) intervention (n = 259). The intervention group was offered community-based 21-day MPRRI program, combining supportive-expressive group, cognitive-behavioral therapy, and Guolin Qigong. QoL was measured using the European Organization for Research and Treatment Quality of Life Version 3 Questionnaire. Multivariable linear models were used to compare changes in QoL values between the two groups.

**Results:**

After adjustment for the QoL score and other covariates at baseline, there was no significant difference in global health status (mean = 3.8, 95% CI − 1.3–9.0, *P *= 0.14) between the two groups after 6 months intervention. While compared with the WLC group, the intervention group showed significant improvements in the QoL score (all *P *< 0.05); however, there were no clinically relevant changes in subscales including emotional functioning (ES = 0.58), cognitive functioning (ES = 0.53), pain (ES = 0.52), physical functioning (ES = 0.36), and insomnia (ES = 0.30).

**Conclusions:**

These preliminary results suggest the MPRRI program is both feasible and acceptable intervention for cancer survivors in community settings and is effective in significant improving QoL above.

## Background

Cancer is the leading cause of mortality in China, accounting for nearly two million deaths each year and approximately one-quarter of all deaths in the country. At the same time, the number of cancer survivors in China is continuously increasing due to advances in early cancer diagnosis and treatment [1–4]. Cancer has been documented as the cause of considerable psychological strain for cancer survivors and their families. Quality of life (QoL) is affected by the need to face a chronic and life-threatening disease and the demands and complications of repeated, lengthy, and often invasive therapies [5–7]. The emotional impact of a cancer diagnosis and treatment is devastating, characterized by shock, anxiety, and depression, and affects daily living [8, 9].

Cancer survivors have a number of psychosocial needs, such as physical, psychological, and social aspects of care and treatment of the disease. These include treatment options and decision-making, the stress on others, sharing the illness experience and connecting with other cancer survivors, and gaining a sense of control [10]. Numerous psychosocial interventions have been developed to evaluate these needs. A psychosocial intervention can be defined as any treatment intended to address the psychological, social, spiritual needs, or any combination of these rather than the disease itself [11]. These interventions commonly incorporate a number of different components but can generally be classified as education, social supportive-expressive group development, guided imaginary, music therapy, cognitive-behavioral techniques, and counseling [12].

In China, after inpatient medical treatment, community health service centers often provide all follow-up care [13], but such care, including psychosocial care, has been shown to be inadequate [14]. Cancer self-help rehabilitation groups are considered one of the most promising aspects of cancer psychosocial interventions, which are multi-factorial programs covering several aspects of well-being and are conducted under real-world conditions, rather than tightly controlled research projects [15, 16]. However, it is not common in China compared with Western countries, and those that were available were ineffective until the first cancer self-help organization, the Shanghai Cancer Rehabilitation Club (SCRC), was formed in 1989. The SCRC is an important survivorship improvement organization, and its goal is to support cancer survivors throughout their care with multi-focused psychosocial interventions.

Although multi-focused psychosocial residence rehabilitation intervention (MPRRI) programs are widely implemented, the effect of such interventions has not been rigorously evaluated. The aim of this study was to evaluate the effects of a 21-day MPRRI program on the QoL of cancer survivors.

## Methods

### Recruitment and study participants

Convenience samples of cancer survivors were collected in Shanghai, China by the SCRC. Participants were primarily recruited through pamphlets and posters around their medical care facilities. The inclusion criteria were: (1) participants had a clinical diagnosis of cancer and showed interest to participate; (2) had completed conventional medical care at a hospital and were medically stable; (3) were between 18 and 70 years old and had an expected survival time of over 1 year; (4) Chinese residents who were able to read, speak, and write Chinese; and (5) had normal cognitive and sensory functions with no psychological condition that might potentially hamper compliance with informed consent.

### Intervention groups

In this study, a total of 388 study participants were enrolled into either a waiting-list comparison (WLC) group (n = 259) or the MPRRI group (n = 129) (November 2013 to January 2015). The WLC group received standard follow-up care as a control, which consisted of health lectures every month and communication with healthcare staff. The intervention group received a multi-focused intervention, consisting of 6 components.

#### (1) Supportive-expressive group

Study participants with same or similar types of cancer were divided into groups and asked to develop a team name and anti-cancer slogan, attend group meetings that were facilitated by SCRC staffs, and focus on expression of emotions in a supportive group environment to reduce negative emotions and promote psychological adjustment [17].

#### (2) Relaxation training and guided imagery

Study participants participated in morning meditation for half an hour and listened to music with guided meditation CD tracks that were designed to promote familiarity with relaxation and visualization. Instructions were provided for CD use, focused breathing, and relaxation techniques. Participants were encouraged to imagine themselves in a peaceful, serene, safe, and secure personal place to rest and let go of their anxiety, worries, or concerns and emerge from their scenario with a sense of feeling refreshed and recharged [18]. This feeling state image of a pleasant and safe scene focused on feelings of peace, calm, and relaxation.

#### (3) Art and music therapy

Art therapy here included music therapy interventions, numerous types of art therapy, and dance/movement therapies [19]. Patients met with a music therapist (a board certified music therapist) for nine 50-min sessions over the course of 21 days. During the session, participants worked in collaboration with rhythmic drumming in a circle under the guidance of the music therapist, who encouraged the participants to release their anxious and tense emotions and help them integrate into one team to facilitate communication with each other [20]. Additionally, patients learned to sing songs, such as “Grateful Heart” with sign language “The Song of the Rehabilitation School” and “Let Love Move,” and dance.

#### (4) Cognitive-behavioral therapy (CBT)

CBT was provided to groups of six to ten individuals for 90 min twice a week for 3 weeks. The therapy included the following validated strategies: stimulus control, confidence restriction, cognitive therapy, and relaxation training. This training was formatted to be consistent with a cognitive-behavioral problem-solving therapy protocol for individual cancer patients. CBT aims to enable participants to effectively solve their personal problems associated with cancer. To this end, they learned to apply self-management skills in striving for personal goals (e.g., in work, household, hobbies, physical activity, family relationships, and social contacts) [21, 22]. Generalization to daily life during and after rehabilitation was facilitated by practicing activities during sessions and by homework assignments (maximally 30 min weekly).

#### (5) Psycho-education and counseling

At the beginning of rehabilitation camp patients watched lectures from Professor Zhaoyou Tang, a famous surgical oncology expert in China, and regularly received some specific topics of health education, such as nutrition education and diet guidance. Many interventions include an educational component to satisfy patients’ needs for comprehensive information about their disease, treatment options, and potentially helpful coping strategies [23, 24].

#### (6) Guolin Qigong

Study participants underwent 2.5 h of Guolin Qigong training once a day for 5 days a week. This training, a type of aerobic exercise, was incorporated to elicit improvements in aerobic capacity [25]. Additionally, the Guolin Qigong training aims to bring the body and mind into balance and to relieve the residual side effects of clinical treatment. This technique places emphasis on meditation and coordinated breathing, together with slow and smooth movements [26]. An instructor and an assistant instructor supervised study participants during each training session.

### QoL scales

QoL was measured by the simplified Chinese version of the European Organization for Research and Treatment quality of life version 3 questionnaire (EORTC QLQ-C30) [27]. The EORTC QLQ-C30 core questionnaire contained five functional scales (physical, role, cognitive, emotional, and social), three symptom scales (fatigue, pain, and nausea and vomiting), a global health status (GHS), a number of single items assessing additional symptoms commonly reported by cancer patients (dyspnea, appetite loss, insomnia, constipation, and diarrhea), and the financial impact of the disease. A high score on the functional scale, the global health status, or overall QoL represents a high or healthy status or a high QoL, whereas a high score for the symptom scale represents a high level of symptomatology or problems. The scoring of the EORTC QLQ-C30 items was performed as stipulated in the EORTC scoring manual [28].

### QoL measurements

Assessments were made at baseline, at 4-week, and 6-month after the intervention in the MPRRI group and at baseline and 4-week after the intervention for the WLC group. The baseline assessment consisted of questionnaires to measure demographic and medical variables, physical activity, and QoL. Similarly, the 4-week and 6-month follow-up questionnaires assessed physical activity and QoL.

### Covariates

We additionally assessed the effects of behavioral and lifestyle factors among intervention groups. The behavioral and lifestyle factors we examined included dietary habits (vegetable and fruit consumption in the past 7 days, having vs. skipping breakfaster each day), smoking status (characterized as current, former, or never smoking), drinking alcohol (including beer, red wine, and white wine consumption as a binary variable) and sleeping status (need to take medicine or not). Additionally, study participants provided information regarding physical activity, such as self-reporting the average time per week spent on which kind of physical activity in the past 4 weeks. Physical activity was defined as those who participate in moderate activities for 30 min, such as jogging, running, bicycling, swimming, table tennis, Qigong, and Taichi. Measurement of physical activity was recorded as binary (Yes or No), binary frequency (≥ 5 times/week or < 5 times/week), and continuous time (hours/week).

### Statistical analysis

The pre-specified primary QoL outcome was GHS on the EORTC QLQ-c30 to test the hypothesis that participants in the 21-day MPRRI group would have better QoL compared with participants in WLC group. Univariable analyses were performed to assess adherence to the assumptions of normality and equal variance, as well as for the detection of outliers. Fisher’s exact or χ^2^ tests were conducted on categorical variables, including sex, marital status, monthly household income per capita, smoking status, cancer type, and medical treatment. Wilcoxon rank test or *t* tests were performed on continuous variables, including age, body mass index (BMI), and years since cancer diagnosis, highest educational achievement, and alcohol consumption.

Multivariable linear regression was used to determine the difference of QoL subscales between two groups at 6 months from baseline. Covariates included sex, age, BMI, education level, monthly household income per capita, cancer type, time since cancer diagnosis, and behavioral and lifestyle factors at baseline.

To explore the effect within groups, we also fit a multilevel linear growth model (MLGM) [29] for subscales. Mean GHS scores and corresponding 95% confidence intervals (CIs) were estimated.

Furthermore, generalized estimating equations (GEE) were used to analyze the change in behavior and lifestyle factors between two groups. Effect sizes (ES) were calculated according to Cohen as indices measuring the magnitude of a treatment effect. An ES < 0.2 reflects “no effect,” 0.2 ≤ ES ≤ 0.5 reflects “small effect,” 0.5 ≤ ES ≤ 0.8 reflects “moderate effect,” and ES ≥ 0.8 reflects “large effect.” [30].

All statistical analyses were performed using SAS version 9.2 (SAS Institute Inc., Cary, NC, US). P values < 0.05 were considered significant.

## Results

### Baseline characteristics

**Fig. 1:**
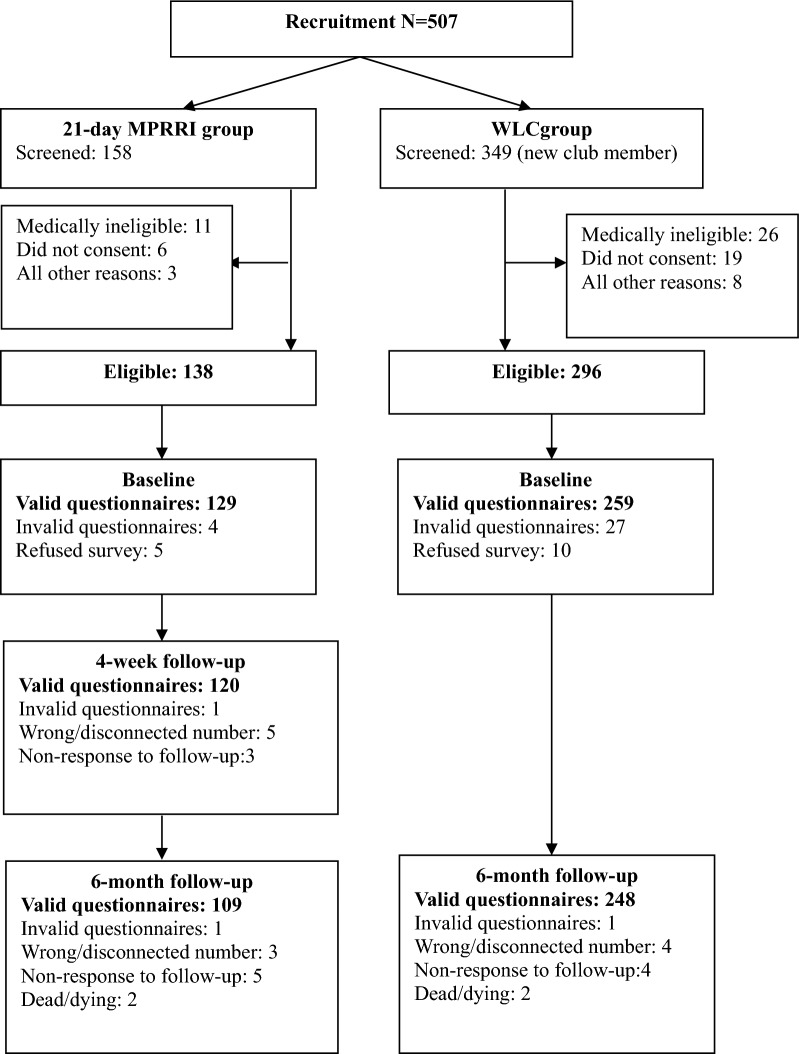
Participant flow chart

An overview of number of patients enrolled in the two groups and lost to follow-up is provided in Fig. 1. The MPRRI group completed 93.02% at 4-week, 86.82% at 6-month, and the WLC group completed 95.75% at 6-month from baseline.

Table 1 shows participants’ baseline characteristics. The groups were balanced in terms of sociodemographic factors, behavior and lifestyle factors, and medical variables. The mean time since initial diagnosis years of participants in the MPRRI group (2.05 ± 1.50 year) was not significantly different than the mean from WLC group (2.32 ± 1.63 year, *P *= 0.09). Lung cancer accounted for 23.3% and 21.2% of the total number of initial cancer diagnoses in the 21-day MPRRI group and WLC group, and breast cancer accounted for 31.8% and 25.9% respectively. There was no significant difference in cancer type between two groups (*P *= 0.63). However, WLC participants were less educated than participants in the 21-day MPRRI group (*P *< 0.0001).

**Table 1:** Sociodemographic, behavior and lifestyle factors, and clinical data at baseline for the intervention groups

Variables	21-day MPRRI	WLC-group	*P*
Age, mean (SD), years	57.0 (7.5)	57.9 (7.2)	0.2296
BMI, mean (SD), kg/m^2^	23.4 (3.4)	23.1 (3.1)	0.4154
Gender, n (%), female	96 (74.4)	173 (66.8)	0.1250
Marital status, n (%)			
Married/with partner	115 (89.2)	227 (87.6)	0.6662
Single/widowed/divorced	14 (10.8)	32 (12.4)	
Education, n (%)			
Compulsory school	31 (24.0)	107 (41.3)	0.001
Apprenticeship/technical college	55 (42.6)	100 (38.6)	
University	43 (33.3)	52 (20.1)	
Monthly household income per capita (Yuan, RMB)			
≤ 2000	22 (17.1)	55 (21.2)	0.3636
2001–4000	87 (67.4)	175 (67.6)	
≥ 4001	20 (15.5)	29 (11.2)	
Current Smoker, n (%)	3 (2.3)	8 (3.1)	0.9187
Drinking alcohol in past 4-week, n (%)	11 (8.5)	11 (4.3)	0.0859
Diagnosis, n (%)			0.6271
Lung cancer	30 (23.3)	55 (21.2)	
Breast cancer	41 (31.8)	67 (25.9)	
Digest tract cancer^a^	32 (24.8)	70 (27.0)	
Gynecological cancer^b^	7 (5.4)	17 (6.6)	
Other type^c^	19 (14.7)	50 (19.3)	
Time since initial diagnosis, mean (SD), years	2.05 (1.50)	2.32 (1.63)	0.0941
Medical treatment, n (%)			
Surgery	107 (82.9)	202 (78.0)	0.2537
Radiotherapy	38 (29.5)	66 (25.5)	0.4050
Chemotherapy	101 (78.3)	189 (73.0)	0.2557
Traditional Chinese medicine	87 (67.4)	154 (59.5)	0.1268

^a^Digest tract cancer include gastric cancer, liver cancer, colorectal cancer, esophageal cancer, cholangiocarcinoma, and pancreatic cancer

^b^Gynecological cancer include cervical cancer, ovarian cancer, vaginal cancer, endometrial cancer, and cancer of vulva

^c^Other type include prostate cancer, nasopharyngeal, laryngeal, thyroid cancer, lymphoma, leukemia, oral cancer, ureteral cancer, bladder cancer, and penile cancer

### Quality of life associations

#### (1) GHS modeling

**Fig. 2:**
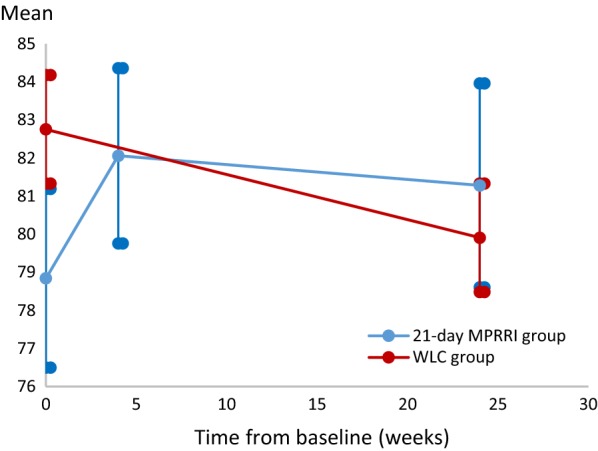
Mean global health status (GHS) scores and corresponding 95% confidence intervals (CIs) were estimated using the model

After adjustment for the QoL score and other covariates at baseline, difference in GHS score between two groups at each of 6-month was not statistically significant (*P *= 0.14) and larger than 10-points, which are considered to be clinically relevant changes in the EORTC QLQ [31] (Table 2). The difference was 3.8 (95% CI, − 1.3–9.0), indicating a greater score in the 21-day MPRRI group, but it was not beyond the 10-ponit threshold for clinical significance. Figure 2 shows the mean GHS scores estimated according to the MLGM model and the corresponding 95% CIs. In both groups, the mean GHS scores tended to improve over time. While GHS (ES = 0.27) showed a small treatment effect.

**Table 2:** Summary of the Quality-of-Life Results

Subscales	WLC		21-day MPRRI		Pre to 6-month between-group change 21 day-MPRRI with WLC		
	Baseline (n = 259)	6-month (n = 248)	Baseline (n = 129)	6-month (n = 109)	∆ (95% CI)	*P*	ES
Global health status	61.1 ± 22.7	63.3 ± 23.2	58.2 ± 22.8	66.7 ± 19.9	3.8 (− 1.3, 9.0)	0.1437	0.2720
Physical functioning	82.7 ± 12.8	80.2 ± 15.4	78.9 ± 15.0	80.8 ± 13.0	3.1 (0.3, 6.4)	0.0332	0.3628
Emotional functioning	84.9 ± 17.1	82.3 ± 19.3	77.1 ± 22.7	83.5 ± 16.4	6.0 (2.5, 9.5)	0.0009	0.5794
Cognitive functioning	84.4 ± 16.2	81.4 ± 16.3	74.2 ± 19.4	79.7 ± 18.8	3.5 (0.1, 6.9)	0.0443	0.5297
Pain	12.9 ± 15.7	15.3 ± 17.9	19.9 ± 22.4	12.3 ± 14.5	− 6.0 (− 9.7, − 2.3)	0.0015	0.5171
Insomnia	19.6 ± 24.1	24.0 ± 26.3	26.4 ± 29.7	22.5 ± 28.8	− 5.3 (− 10.2, − 0.4)	0.0346	0.3011

#### (2) Other scales

Among the other scales of the EORTC QLQ-C30, the 21-day MPRRI intervention group showed significant improvements over the WLC group (*P *< 0.05) in *emotional functioning* (ES = 0.58), *cognitive functioning* (ES = 0.53), *pain* (ES = 0.52), *physical functioning* (ES = 0.36), and insomnia (ES = 0.30) (Table 2), with the former 3 subscales representing moderate treatment effect. These improvements, however, did not reach the predetermined minimal clinically important difference of 10 points. No significant differences were observed for scores on the other scales (*role functioning*, *social functioning*, *fatigue*, *nausea* or *vomiting*, *dyspnea*, *appetite loss*, *constipation*, *diarrhea*, *financial difficulties*).

#### (3) Intra-group effect

Improvements within the 21-day MPRRI group and GHS were both significant (*P *< 0.0001) and clinical improvement (estimated mean difference = 11.2, 95% CI 7.0–15.5) at 4-week from baseline, but not continuing to the 6-month values. *Physical functioning, emotional functioning*, *cognitive functioning*, and *pain* were significant (*P *< 0.05) at 4-week from baseline and continued until 6-month follow-up, except for *physical functioning* (Table 3). In contrast, the WLC group exhibited significant declines from baseline in *physical functioning, emotional functioning*, *cognitive functioning* (*P *< 0.0001), *insomnia* (*P *< 0.01), and *pain* (*P *< 0.05).

**Table 3:** Effects of QoL within the two groups’ cancer survivors among three assessments

	Pre to 4-week change within 21-day	Pre to 6-month change within 21-day	Pre to 6-month change within WLC
Physical functioning	3.2 (− 1.2, 2.8)**	2.4 (− 0.2, 5.1)	− 2.8 (− 4.7, − 1.0)**
Emotional functioning	8.8 (5.5, 12.1)***	6.7 (3.7, 9.7)***	− 3.0 (− 5.1, − 0.9)**
Cognitive functioning	4.6 (1.7, 7.5)**	5.5 (2.6, 8.4)**	− 3.1 (− 5.1, − 1.2)**
Global health status	11.2 (7.0, 15.5)***	8.4 (3.8, 13.0)**	2.2 (− 1.1, 5.4)
Pain	− 5.8 (− 9.1, − 2.5)**	− 7.6 (− 10.9, − 4.2)***	2.6 (0.3, 4.9)*
Insomnia	− 0.8 (− 4.6, 3.1)	− 3.2 (− 7.5, 1.0)	4.4 (1.5, 7.3)**

*** < 0.0001, ** < 0.01, * < 0.05

### Changes in behavior and lifestyle factor between two groups

Using GEE to analyze the changes in behavior and lifestyle factors between two groups, we observed a significant interaction between time and group for not only the proportion of physical activity participation (OR = 2.042, 95% CI 1.139–3.663) but also in the frequency of physical activity (OR = 1.757, 95% CI 1.330–2.332) (Table 4). Additionally, the change in the amount of time spent in physical activity from baseline to 6 months between groups was found to be significant (mean difference = 4.54, 95% CI 3.19–5.90) (Table 4).

**Table 4:** Comparison of the change in behavior and lifestyle factors between the two groups (n, (%))

	WLC-group		21-day MPRRI group			*P* value
	Baseline	6-month	Baseline	4-week	6-month	
Physical activity						
Participate in	166 (64.1)	192 (80.0)	101 (78.3)	115 (95.8)	107 (95.5)	0.0166
≥ 5 times/week	99 (38.2)	110 (45.8)	63 (48.8)	90 (75.0)	88 (78.6)	< 0.0001
Time(hours/week), mean (SD)	3.7 (5.4)	4.8 (5.4)	5.2 (6.7)	10.8 (6.8)	10.5 (8.4)	< 0.0001
Have breakfast everyday	252 (97.3)	229 (95.4)	124 (96.1)	117 (97.5)	111 (99.1)	0.0751
Intake fruits everyday	192 (74.1)	177 (73.7)	106 (82.2)	101 (84.2)	95 (84.8)	0.5653
Vegetable intake ≥ 250 g/day	100 (38.6)	97 (40.4)	65 (50.4)	58 (48.3)	66 (58.9)	0.3942
Sleep medication	68 (26.3)	65 (27.1)	42 (32.6)	30 (25.0)	28 (25.0)	0.0814

## Discussion

In this study, cancer survivors in the 21-day MPRRI group exhibited improvements in most of the subscales of QoL, whereas the QoL of the participants in WLC group continuously declined. Moreover, the mean difference of subscales from baseline to 6-month follow-up in the 21-day MPRRI group showed greater advancement than that of the WLC group.

The SCRC offered continuous training courses for 21 days in one session on how to confront the disease, how to deal with emotions, how to interact with others, and how to plan for the future. Furthermore, the provision of factual information, development of a sense of community, transformation in terms of personal change and spiritual growth, and a feelings of empowerment at individual and community levels were also considered important. Possible mechanisms of intervention include maximum possible acquisition of various support care and resources.

### Emotional support

Cancer survivors experience physical, psychological, mental, and social problems beginning with their diagnosis [8, 9]. According to research reports, emotional support for cancer patients living in China is heavily dependent on family and otherwise relatively limited [32]. Additionally, family caregivers also experience great stress [17, 33]. During this intervention, cancer patients can receive further emotional support from SCRC staff and from cancer survivors with similar experiences.

### Information support

A lack of information may produce feelings of uncertainty and can impede decision-making [34, 35]. Patient care following cancer treatment and quality of life could be improved by providing ongoing education about lifestyle factors related to cancer risk, disease surveillance, and resources for treating and coping with cancer [36] and help develop community cancer support care to meet cancer survivors’ needs for accurate information [35]. In the 21-day MPRRI group, information support from both peer-patients and clinical experts within the same type of cancer in the form of communications seminars was found to help cancer survivors address their dilemmas and cope with their difficult situation.

### Social recognition

Most cancer patients suffered from discrimination, including both feelings of discrimination and their actual experience. If they are discriminated against by people around them, they might become more vulnerable with low self-esteem [37, 38]. The participants will get much social recognition by from SCRC, as self-support service, even after intervention.

Although participants had no significant improvements in the GHS/QL, there were improvements in both the functioning (emotional, cognitive) and symptom (pain) scales after the intervention, which suggests that the rehabilitation course for cancer survivors’ overall QoL, improvement in functional areas, and symptom relief is effective.

Cognitive and emotional dysfunction in cancer survivors may partly be psychosocial consequences associated with a chronic illness including a death threat [8]. For functioning scales, cancer survivors in the intervention group may benefit from CBT [21], art and music therapy [20], and supportive-expressive group [17], which seem to positively affect cancer patients’ emotional functioning, such as anxiety, depression, coping stress, anger and mood, [17, 19]. Similarly, MPRRI program provided the cancer survivors with CBT and psycho-education and counselling [17], which focuses on recognizing and changing maladaptive thoughts and behaviors, and focuses on modifying problems with accuracy information from a qualified professional, and result in improving the cognitive functioning. For symptom scales, as noted previously, relaxation training and guided imagery and Guolin Qigong are a series of techniques not only using mental imagery but also physical activities to facilitate relaxation [17], which can potentially contributes to disorder symptoms, such as pain, fatigue and sleep problems [18, 26].

This MPRRI includes six different component intervention measures, which are worked in collaboration with each other and have comprehensive effectiveness. The non-pharmacologic supportive strategies or combine self-management with group peer support could promote QoL in cancer survivors related fatigue, meet the cancer patients supportive care needs and psychological distress [39, 40]. The psycho-education and counseling targets social support, and explains how life events and the social environment affect mood, the influence of mood on social functioning, and provides normalization and validation of participants’ experiences and reactions to cancer [41].

In addition, one pronounced improvement in the MPRRI group was physical activity. After the intervention, there were great changes to not only to the proportion of attending physical activity and on the frequency of physical activity per week, but also on physical activity time for both groups. These changes, however, were much more pronounced in the MPRRI group than the WLC group. Historically, clinicians advised cancer patients to rest and to avoid physical activity; however, emerging research on exercise has challenged this recommendation [42]. In 2012, the American Cancer Society (ACS) released guidelines for cancer patients and survivors promoting physical activity to improve cancer outcomes [43], emphasizing regular physical activity of moderate intensity (> 3 h/week) is safe and may have improvements in physiology, body composition, physical functions, psychological outcomes [44], quality of life and cancer-related fatigue in cancer progression [45, 46] and survival [47, 48]. However, fewer than half of cancer survivors are meeting the official guidelines for physical activity [48]. One is that the promotion of physical activity is not integrated into routine clinical practice, which can cause the cancer survivors to overlook physical activity after treatment. This is due to lack of evaluation of the feasibility and effectiveness physical activity and limited resources [48, 49]. As such, it is crucial to develop and evaluate new strategies to increase access to and maintenance of physical activity and to improve quality of life in this dramatically growing population of cancer survivors.

In this study, we developed a totally new concept and rehabilitation of physical activity-Guolin Qigong, in a real-life setting; it is relaxation training combined with sustainable physical activities.

The MPRRI program is a group-based intervention to facilitate deeply communication among cancer survivors, even beyond this program they can exchange the experience of the course and a new life. Additionally, this program is a multi-focused psychosocial intervention, combining the modern science discipline including psychological components, and traditional Chinese medicine including Guolin Qigong, which is a feasible measure for cancer survivors. As the implementers of this program, the SCRC staffs themselves were also cancer survivors. Their identities were helpful to narrow the psychological gap between themselves and the participants. Moreover, their experience in cancer diagnosis, treatment and rehabilitation can be transferred directly to the participants. Of note, this study supports the feasibility of the MPRRI program in China, which provides a new insight and may be generalizable to other countries and geographical regions.

In our study, the significant changes in some subscales of QoL from the 4-week to 6-month points within follow-up showed non-continuous progress, especially in symptom domains. Continuity of care should be considered a core element of high-quality primary care in cancer rehabilitation stage after the MPRRI.

### Strengths and limitations

To the best of our knowledge, this is the first study to evaluate the effect of combined supportive-expressive group, guided visualization, cognitive-behavioral therapy, music therapy, psycho-education, and Guolin Qigong on cancer survivors’ QoL. While this study has several limitations. First, the results presented in this analysis were based on recruited participants rather than a random sample. There is likely to be selection bias in both groups. For example, these study participants may be healthier than is generally the case for patients with cancer. Second, although the proportions of different types of cancer between the two groups showed a reasonably good balance, we did not obtain the data on their specific pathological cancer type with respect to stage or complex clinical treatment, such as stage of cancer (early or late stage), different type of chemotherapy, radiotherapy, surgery or other treatment, or co-morbid chronic diseases. These factors may have an impact on the effectiveness of interventions. Third, because of the multi-component nature of the intervention, it is not possible to conclude which component was truly effective. Additionally, as an exploratory study, many endpoints were analyzed and some reached significance by chance (type I error), which makes it difficult to draw firm conclusions. Fourth, a response shift may have occurred if a patient changed his or her internal reference base of health status over time as a consequence of his or her cancer experience and mask true changes. Fifth, the role of optimism may be a significant issue in this study. Some people always have a positive health response and hide their infirmity or overestimate their true health status. These biases may have had the effect of inflating QoL scores beyond what would otherwise be recorded. Further, the lack of equilibrium mean score of QoL between the two groups at baseline could cause confounding effects and bias. Further studies should focus on changes in patients’ life behaviors and the long-term effects of the interventions. More medical data of the cancer survivors in the two groups, such as, the stage of cancer, clinical treatment, co-morbid chronic diseases, can be collected and compared in parallel or stratified analysis can be made at each time points.

## Conclusions

These preliminary results suggest a combined intervention program is both feasible and acceptable intervention for cancer survivors in community settings and is effective in significant improving QoL above (emotional functioning, cognitive functioning and pain) without clinical change.

## Abbreviations

MPRRI: multi-focused psychosocial residence rehabilitation intervention
SCRC: Shanghai Cancer Rehabilitation Club
WLC: waiting-list comparison
QoL: quality of life
EORTC QLQ-C30: European Organization for Research and Treatment Quality of Life Version 3 Questionnaire
CBT: cognitive-behavioral therapy
GHS: global health status
BMI: body mass index
MLGM: multilevel linear growth model
CIs: confidence intervals
GEE: generalized estimating equations
ES: effect sizes
ACS: American Cancer Society
